# Drainage lysimeter based measurement of water requirement and crop coefficient of bread wheat under semi-arid climate of Melkassa, Ethiopia

**DOI:** 10.1016/j.heliyon.2024.e36969

**Published:** 2024-08-27

**Authors:** Gobena Dirirsa Bayisa, Mekonen Ayana, Boja Mekonnen, Tilahun Hordofa, Megersa Olumana Dinka

**Affiliations:** a Adama Science and Technology University, Department of Water Resources Engineering, Adama, Ethiopia; b Ethiopian Institute of Agricultural Research, Melkassa Research Center, Adama, Ethiopia; c Department of Civil Engineering Science, University of Johannesburg, Johannesburg, South Africa

**Keywords:** Crop coefficient, Drainage lysimeter, Evapotranspiration, Semi-arid, Wheat

## Abstract

In view of the changing climate and growing global food demand, efficient water management is crucial for sustainable agriculture. Accurate measurement of evapotranspiration is essential for determining crop water demand and consequently for designing and managing irrigation systems. This study, conducted at Melkassa Agricultural Research Center in Ethiopia, utilized a drainage lysimeter to investigate the water requirements and crop coefficients of the Kingbird wheat variety during the December to March cropping season in 2021/22 and 2022/23. The experiment involved planting Kingbird wheat both inside and outside the lysimeter and irrigating using a watering can. Neutron probe measurement monitored the water balance in the soil. The study determined an average crop evapotranspiration of 427.28 mm and a reference evapotranspiration of 471.30 mm indicating a water requirement of 4273 m³ to fully grow wheat on a hectare of land. The derived average crop coefficient values were 0.43, 0.93, 1.15, and 0.30 for the initial, mid-season, and end growth stages, respectively. Furthermore, a fifth-order polynomial function was developed to predict crop coefficient values based on days after sowing. The findings provide valuable insights for enhancing the design and management of irrigated wheat production in the region. The specific crop coefficient values determined for different growth stages are crucial for optimizing irrigation scheduling and improving water-use efficiency, contributing to sustainable wheat production in semi-arid environment.

## Highlights

• A drainage lysimeter was utilized to determine the water requirements and crop coefficients for the Kingbird wheat variety in a water-scarce region of Ethiopia.
• The Kingbird wheat variety requires 4273 m³ of water per hectare for the entire growing period.
• Actual derived specific crop coefficient values for the crop are 0.43, 0.93, 1.15, and 0.30 for the initial, mid-season, and end growth stages, respectively.
• A fifth-degree polynomial function was developed to predict crop coefficient of wheat based on days after sowing.

## 1 Introduction

Climate change, increased water demand and environmental degradation are reducing water supply for irrigated agriculture in arid and semi-arid areas. Despite these challenges, it is essential to increase agricultural productivity in order to address global food demands [1]. As a critical response to growing water shortage in agriculture, boosting water productivity in rain-fed and irrigated agriculture lowers the need for extra water [2]. Effective irrigation management rely on accurate irrigation scheduling to meet the specific water demands of the crops. Water requirements for various crops depend mainly on evapotranspiration which is affected by weather parameters, management, environmental factors, crop types and their growing stages. Moreover, water requirements for the same crop differ depending on the crop variety, agro-metrological zone, irrigation method and management [3].

Estimate of both the crop evapotranspiration (ETc) and reference evapotranspiration (ETo) is indispensable for determining irrigation scheduling, irrigation system designs, and hydrological studies [4]. A widely recognized method for predicting crop water requirements for irrigation scheduling is outlined in the FAO's Irrigation and Drainage Paper No. 56 [4]. This method relates the ETc, to that of ETo, by considering appropriate crop coefficient (Kc). The standard values of Kc for various crops are given by Allen et al. [4]. However, a local calibration of the Kc values is essential for precise water management before utilizing them for estimating ETc [5]. Water balance experiments employing lysimeter of different types are performed to measure actual estimates of ETc [6]. Lysimeters represent an accurate tool in the determination of the water balance components in the soil–plant–atmosphere system, representing the real field conditions [7]. It measures the amount of water loss due to drainage, transpiration and evaporation from cropped lysimeters, which is then used to calibrate the Kc values [8,9]. Area-specific Kc are then developed for predicting ETc from ETo.

The present study was conducted in the semi-arid region of the Awash Basin in Ethiopia, an area known for its scarce water resources and inefficient irrigation water management. With water demand surpassing supply, the basin is currently experiencing significant water stress [10]. This region faces an escalating challenge of water insecurity [11], highlighting the critical importance of precise irrigation management. This issue is further exacerbated by the Ethiopian government's recent commitment to increasing agricultural production, primarily through the expanded use of irrigated wheat [12], placing additional strain on the already limited water resources.

Some research studies have investigated wheat water use in Ethiopia. Ketema et al. [13] provided insights into crop water requirements and crop coefficients for Kekeba wheat variety under rainfed conditions during the main rainy season. Conversely, Amdneh and Ayana [14] focused on durum wheat, specifically the Utuba variety, in sub-moist climatic conditions. The current study concentrates on the Kingbird variety, a multi-disease resistant cultivar that consistently outperforms other varieties like Ogolcho, Biqa, and Kakaba in terms of yield and resilience, making it a promising choice for farmers in stem rust-prone regions of Ethiopia [[15], [16], [17]]. Kingbird's early maturity, high yield, and adaptability to local conditions make it well-suited for low-to mid-altitude wheat-growing areas [15]. Despite its advantages, information on the Kingbird wheat variety's crop water requirement and coefficient characteristics are lacking, which are important for better irrigation water management. Additionally, in contrast to earlier research, this study focused on the dry season, which is a time when there is less water available, highlighting the particular difficulties associated with water shortage in the area.

Therefore, this study aimed to determine water requirements and crop coefficient of the Kingbird wheat variety under irrigated conditions in the water-limited semi-arid Awash basin, Ethiopia, to support effective irrigation water management in the region.

## 2 Materials and methods

### 2.1 Description of the study area

**Fig. 1:**
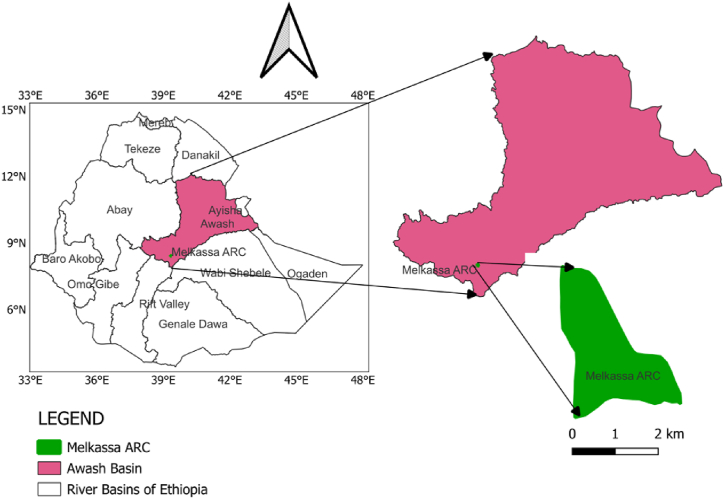
Study area map.

The field experiment was conducted at Melkassa Agricultural Research Center (MARC), in the experimental fields under drainage lysimeters for two consecutive years, from December to March of 2021/22 and 2022/23, aligning with the main wheat irrigation season characterized by limited rainfall and significant water scarcity. This timing allowed to evaluate water requirements and crop coefficient, providing insights into optimizing water use and improving strategies for regions with similar climatic conditions. The site is located at an elevation of 1550 m above sea level 8°24′ N latitude and 39°21′ E longitude in the Awash basin of Ethiopia (Fig. 1). The area is characterized by semi-arid tropical climatic conditions with two distinct seasons. The wet season occurs mainly between June and September while the dry season is experienced during the remaining months of the year with few rain events from March to May. The climatic variables were monitored by the weather station of the research center located near by the lysimeter plots. The long-term (1992–2022) average daytime temperature recorded in the area was 29 °CC, and the average nighttime temperature was 14 °C. The area's average relative humidity is 53.66 %, and its average wind speed is 2.64 m s^−1^.

**Fig. 2:**
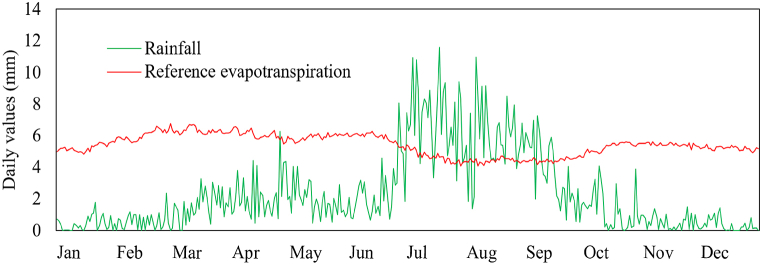
Comparison of rainfall and evapotranspiration illustrating potential water deficits in the study area.

Fig. 2 displays the recorded long-term seasonal rainfall and computed reference evapotranspiration using the FAO Penman Monteith equation, providing insights into the area's water budget. The data highlights a significant rainfall deficit and high evapotranspiration demand throughout the year, except for the period between late June and early September. This underscores the critical need for irrigation in agricultural production, particularly during the dry seasons. The long-term (1992–2022) rainfall analysis, presented in Fig. 2 and Table 1, reveals distinct seasonal variations. Peak rainfall occurs in July and August, with an average annual rainfall of 845.5 mm and a standard deviation of 148.9 mm indicating low annual variability with a 17 % coefficient of variation [18]. Notably, 56 % of the annual rainfall falls during the summer months (June, July, and August) with moderate variability, while the winter months (December, January, and February) received only 5 % of the annual rainfall, with high variability exceeding 90 %. These findings highlight the substantial seasonal variations and the need for effective irrigation water management practices during the dry seasons to optimize the use of available water resources.

**Table 1:** Monthly, seasonal and annual rainfall analysis in the study area from 1992 to 2022.

Month /Season	Minimum (mm)	Maximum (mm)	Mean (mm)	Standard Deviation	Coefficient of variation (%)	Sen's slope	p-value
January	0.0	53.0	12.9	17.9	138.5	0.00	0.280
February	0.0	122.8	16.4	26.0	159.3	0.00	0.571
March	0.0	151.8	48.4	41.1	84.9	−0.51	0.696
April	0.0	267.1	65.3	51.2	78.5	0.35	0.646
May	0.0	150.7	57.0	44.0	77.2	1.05	0.308
June	4.0	184.0	72.9	38.0	52.2	−0.17	0.812
July	71.7	453.9	221.5	96.4	43.5	0.53	0.812
August	80.8	325.7	181.5	60.3	33.2	−0.64	0.610
September	34.0	200.9	105.7	44.6	42.2	1.00	0.277
October	0.0	146.0	38.8	44.3	114.3	−0.29	0.366
November	0.0	74.7	14.7	18.5	125.9	0.26	0.088
December	0.0	57.6	10.4	16.3	156.7	−0.19	0.068
Spring	52.5	288.9	159.3	61.3	38.5	0.25	0.812
Winter	0.0	126.0	39.8	36.0	90.8	−0.90	0.174
Fall	23.0	309.7	170.6	72.5	42.5	1.42	0.314
Summer	241.0	816.8	475.9	121.2	25.5	0.24	0.973
Annual	477.3	1093.1	845.5	148.9	17.6	3.83	0.208

The seasons are categorized as follows: Spring includes September, October, and November and is a transitional period; Winter, encompassing December, January, and February, is a dry season; Fall, consisting of March, April, and May, represents a short rainy season; and Summer, which spans June, July, and August, is the main rainy season. A positive value of Sen's slope indicates an increasing trend in the time series, while a negative value denotes a decreasing trend.

**Fig. 3:**
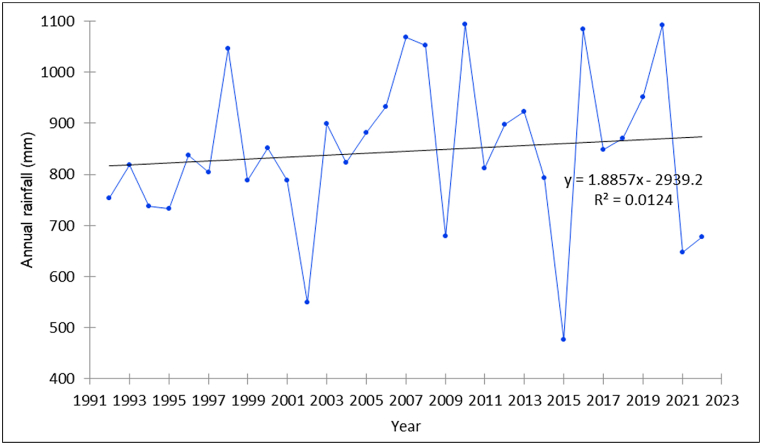
Rainfall pattern of the study area (1992–2022).

The Mann–Kendall and Sen's estimator trend test for rainfall showed a statistically significant decreasing trend for December. Non-significant decreasing trends were observed for March, June, August, and October (at the 10 % level of significance) (Table 1). Conversely, November exhibited a statistically significant increasing trend, while non-significant increasing trends were noted for April, May, July, and September. January and February showed no significant trend. Additionally, the dry season (December, January, and February) had a non-significant decreasing trend, while other seasons exhibited non-significant increasing trends. Overall, the study area displayed a non-significant increasing annual rainfall trend, with a rate of 1.89 mm/year (Fig. 3). This emphasizes the importance of strategic irrigation management to address the challenges posed by water scarcity during the dry season.

**Fig. 4:**
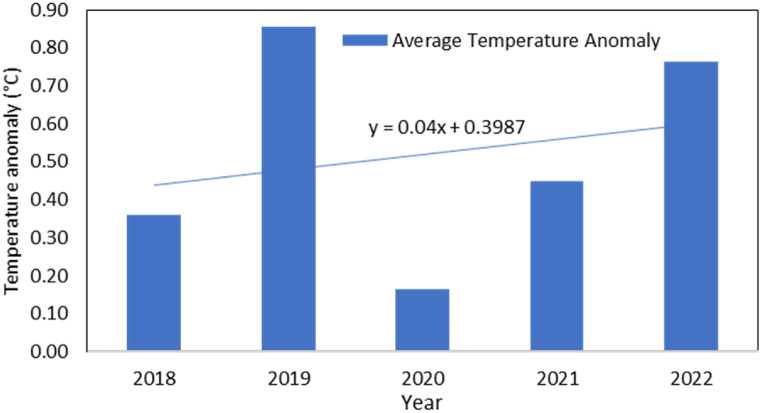
Annual Mean temperature anomaly relative to the 1988–2017 baseline.

Fig. 4 shows a warming trend of about 0.4 °C per decade over the past 30 years in the study area. This increase in temperature can lead to higher water demand for crops, shift growing seasons, and increase the risk of heat stress. These changes highlight the need for improved water management strategies to adapt to the warming climate.

The soil characteristics of the lysimeter, as assessed by the MARC soil laboratory, are presented in Table 2. Soil particle size distribution was determined using the Bouyoucos hydrometer method [19]. Water holding capacity at Field Capacity (FC) and Permanent Wilting Point (PWP) was measured using a pressure plate method [20]. Bulk density (BD) was calculated from undisturbed core samples [21]. Soil pH and electrical conductivity (EC) were measured in a supernatant suspension of a 1:2.5 soil-water mixture using a pH meter [22], and a conductivity meter, respectively. Total nitrogen content was determined using the Kjeldahl procedure [23]. The available water content (AWC) in millimeters was computed by subtracting the PWP from the FC and multiplying the result by the BD and the depth.

**Table 2:** Lysimeter soil characteristics.

Soil depth (cm)	Texture	FC% (w/w)	WP% (w/w)	AWC (mm/15 cm)	BD (g cm^−3^)
0–15	Clay loam	31.80	20.89	18.33	1.12
15–30	Clay loam	32.62	22.22	17.47	1.12
30–45	Clay loam	32.89	22.33	17.90	1.13
45–60	Clay loam	32.07	22.41	16.52	1.14
60–90	Clay loam	32.05	21.74	17.54	1.14

FC and WP are soil moisture contents at field capacity and wilting point in weight basis, AWC is available water content in mm per 15 cm soil depth, and BD is the soil bulk density.

The laboratory results revealed that the soil in the study area is classified as clay loam. The soil's water content at field capacity in weight basis ranged from 31.80 to 32.89 %, and the permanent wilting point ranged from 20.89 to 22.41 %. The bulk density is in the range of 1.12–1.14 g cm^−3^. The average soil pH and electrical conductivity (EC) of the lysimeter plots determined were 6.50 and 1.64 dS m^−1^, respectively, while the total nitrogen content determined was 0.09 %.

### 2.2 Lysimeter experimental setup

**Fig. 5:**
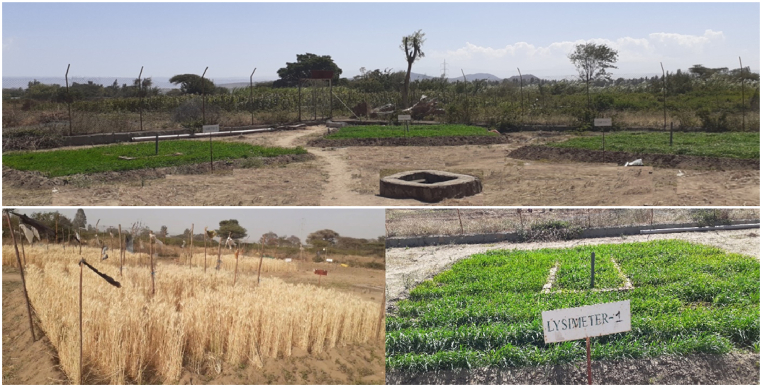
Layout of the experimental lysimeter and visuals of field condition.

The lysimeter experiment was conducted to determine the ETc and drive Kc for the wheat crop. The layout of the experimental lysimeter plots is depicted in Fig. 5. Three non-weighing drainage lysimeters were used in both years (2021-22 and 2022-23) in the experimental station. The lysimeter plots are positioned centrally within a 6 m × 6 m area where wheat was cultivated. Among them, two lysimeter plots measure 2 m in length and width, with a soil depth of 1 m each, while one lysimeter plot is 2 m long, 1 m wide, and also has a soil depth of 1 m. The crops inside and outside the lysimeter were grown and treated in a similar way to reduce edge effects. The lysimeters are furnished with access tubes designed for neutron probe readings, along with an aeration vent and drainage chamber connected to underground steel pipes for the purpose of draining excess water percolated beyond the depth of the root zone. The concrete walls with plastic lining surrounding the lysimeter plots prevents the lateral flow of water in and out of the system. To prevent runoff from entering or leaving the lysimeter, the rim was maintained 0.10 m above the surface level. The water applied to the lysimeters was measured with a calibrated watering can.

### 2.3 Crop management

The Kingbird bread wheat cultivar was sown at a density of 125 kg ha^−1^ on December 10, 2021, for the first-year experiment and December 13, 2022, for the second-year experiment. The lysimeter plots were prepared with a 60 cm ridge spacing, and a single row of seeds was sown on each side of the ridge, spaced at 20 cm. Urea (46 % N) and NPS (19 % N, 38 % P_2_O_5_, and 7 % S) fertilizers were used as sources of nutrient to enhance the growth and yield of wheat plants. A full dose of NPS (125 kg ha^−1^) and 25 % of Urea (25 kg ha^−1^) were applied as a basal dressing at the time of planting, while the remaining urea dose (75 kg ha^−1^) was top-dressed at tillering. The crop was harvested on March 21, 2022, during the 2021-22 cropping season and on March 26, 2023, during the 2022-23 cropping season. The entire crop duration was divided into initial, development, mid-season, and late season stages [24]. Table 3 gives the details of the crop growth period during the cropping season.

**Table 3:** Detail of the crop growth period and growth stages.

Crop	Crop cultivar	Sowing date	Physiological maturity date	Growth stage^a^ (days)			
				I	D	Md	LS
Wheat (Triticum aestivum L.)	Kingbird	December 10, 2021	March 14, 2022	17	22	38	18
		December 13, 2022	March 19, 2023	17	22	40	18

a I, D, Md, and LS correspond to the initial, development, mid-season, and late-season growth stages, respectively.

### 2.4 Soil moisture monitoring

Soil moisture content were monitored from the lysimeter plots at an interval of 15 cm to a depth of 90 cm before and after every irrigation. The measurement of volumetric soil water content at depths from 15 cm to 90 cm in the lysimeter was conducted using a CPN503 neutron moisture meter. Soil moisture content for the top 0–15 cm soil layer was monitored by the gravimetric method, and converted to volumetric water content by multiplying with the soil bunk density using Eq (1) [25].

[1] $$\theta_{v}=\frac{\left(M_{s}-M_{d}\right)}{M_{d}}\times \frac{\rho_{b}}{\rho_{w}}$$

Where $\theta_{v}$ is volumetric water content (cm^3^ cm^−3^), $M_{s}$ is the mass of the soil immediately after it is sampled, $M_{d}$ is the mass of the soil after drying, $\rho_{b}$ is bulk density of the soil (g cm^−3^), and $\rho_{w}$ is density of water typically assumed to be 1 g cm^−3^.

### 2.5 Irrigation management

The irrigation management strategy employed in the lysimeter aimed to establish optimal soil moisture conditions by maintaining the depletion fraction, p-value, at or below 0.4. This was achieved through regular irrigation intervals, typically ranging from 3 to 5 days, contingent upon the observed rate of soil water loss. The depth of water needed to re-fill the soil to the field capacity was calculated using Eq (2).

[2] $$D=\left(\frac{\theta_{FC}-\theta_{BI}}{100}\right)\times Zr$$

Where: D is depth of water required to irrigate (mm), $\theta_{FC}$ is volumetric water content at field capacity (%), $\theta_{BI}$ is volumetric water content of soil before irrigation (%), Zr is plant root depth considered for irrigation (mm).

### 2.6 Actual evapotranspiration

The determination of water balance components from lysimeter study was employed to estimate the actual evapotranspiration (ETa) of the crop. For the water balance analysis, the depth of irrigation water applied, soil moisture depletion from the respective root zone depth for different periods between two successive soil sampling, and the percolated excess water measured from the drainage collection chamber of the Lysimeter were used. The estimated value by water balance equation was considered as actual ET [26], and computed as described in Eq (3).

[3] $$ETa=\left[\frac{\left(I+P-D\pm \Delta S\right)}{\Delta t}\right]$$

Where: ETa is actual evapotranspiration, I is irrigation depth in mm, P is precipitation in mm; D is deep percolation in mm, ΔS is soil moisture storage change in mm, and Δt is the time interval between two consecutive measurements in days.

The USDA-SC method within FAO-CROPWAT 8 model [27] was used to compute effective rainfall during the field experimentation.

### 2.7 Reference evapotranspiration

FAO-CROPWAT 8.0 model [27] was used to calculate the ETo with the daily time step employing modified FAO Penman–Monteith equation [4]. Weather station of Melkassa Agricultural Research Center which is located about 50m from the lysimeter plot was used as data source of recorded weather data during the growing season.

### 2.8 Crop coefficient

The Kc, values reflect the relative water consumption capacity of a specific crop during the different growing stages. Kc was calculated using lysimeter measurements, as the ratio of ETc, derived from water balance method, to ETo, as estimated by FAO-CROPWAT 8 model, following Eq (4).

[4] $$Kc=\frac{ETc}{ETo}$$

Where: Kc is crop coefficient (dimensionless); ETc is crop evapotranspiration (mm day^−1^) and ETo is reference evapotranspiration (mm day^−1^).

The determined Kc values were compared with FAO recommended values for the crop [4].

## 3 Results and discussion

### 3.1 Weather conditions during the study period

**Fig. 6:**
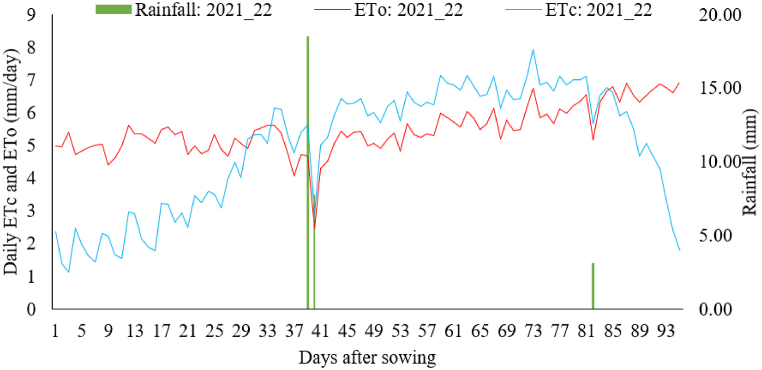
Reference evapotranspiration, crop evapotranspiration and rainfall during 2021/22 season.

**Fig. 7:**
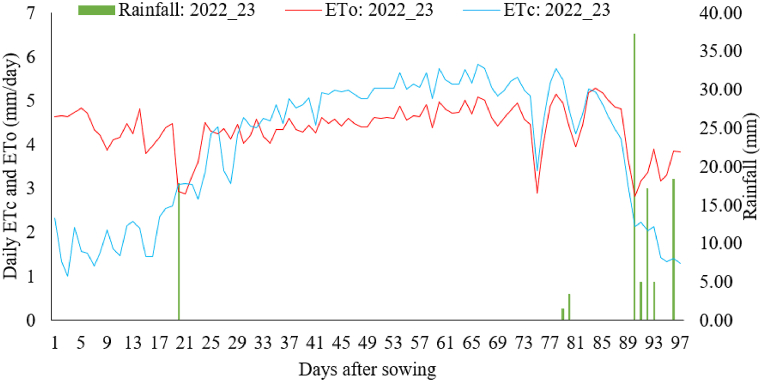
Reference evapotranspiration, crop evapotranspiration and rainfall during 2022/23 season.

The important meteorological factors contributing to the calculation of reference evapotranspiration in both the 2021/22 and 2022/23 growing seasons are outlined in Table 4, summarized over ten-day intervals. During the 2021/22 growing period, the minimum air temperature ranged from 10.0 to 16.1 °C, and the maximum temperature ranged from 28.7 to 32.5 °C. The relative humidity varied between 41.1 and 52.1 %, with an average of 48.1 %. The average wind speed was 2.4 m/s, and the recorded average daily sunshine hours were 9.5. During the 2022/23 growing period, the minimum night air temperature ranged from 8.3 to 14.4 °C, while the maximum temperature varied between 27.3 and 30.7 °C (Table 4). The relative humidity ranged from 63.3 to 84.9 %, with an average of 77.9 %. The average wind speed was 2.4 m/s, and the recorded average daily sunshine hours were 8.5 h. There were few instances of rainfall during the field experiments as shown in Fig. 6, Fig. 7.

**Table 4:** Average of ten-day weather data during the growing season of 2021/22 and 2022/23 at the experimental site.

DAS	2021/22 growing season					2022/23 growing season				
	Tmin (°C)	Tmax (°C)	RH (%)	U_2_ (m s^−1^)	Sunshine (hr)	Tmin (°C)	Tmax (°C)	RH (%)	U_2_ (m s^−1^)	Sunshine (hr)
1–10	10.0	28.2	49.2	2.3	9.6	8.7	28.6	63.3	2.2	9.1
11–20	13.2	29.7	50.6	2.7	9.8	12.4	27.8	66.6	2.6	6.8
21–30	15.4	30.1	52.1	2.2	8.9	9.9	27.4	83.1	2.5	9.3
31–40	12.0	30.1	50.6	2.1	8.3	9.6	28.0	82.9	2.9	10.1
41–50	12.4	28.7	51.7	2.2	9.5	11.7	30.2	84.9	2.2	10.0
51–60	9.4	28.7	46.8	2.5	9.9	8.3	29.9	83.8	2.6	10.1
61–70	12.4	30.5	47.8	2.4	9.9	9.4	29.3	82.5	2.9	10.1
71–80	13.2	32.2	46.6	2.5	9.6	11.2	30.7	81.2	1.9	8.1
81–90	13.9	32.1	44.2	2.7	10.0	13.9	30.3	80.6	2.7	7.3
91–100	16.1	32.5	41.1	2.6	9.6	14.4	27.3	70.1	1.6	3.9
**Average**	**12.8**	**30.3**	**48.1**	**2.4**	**9.5**	**10.9**	**28.9**	**77.9**	**2.4**	**8.5**

DAS is days after sowing, Tmin is minimum temperature, Tmax is maximum temperature, RH is relative humidity, and U₂ is the wind speed at a height of 2 m.

### 3.2 Evapotranspiration of wheat

The daily variation in crop evapotranspiration throughout the growth period is illustrated in Fig. 6, Fig. 7. During the initial stages, the values were lower, but they increased during the vegetative growth stage, peaked during the reproductive and grain filling stage, and gradually declined during the maturity stage. Table 5 presents the average evapotranspiration for each growth stage: initial, development, mid-season, and late-season. The pooled values ranged from a minimum of 1.90 mm day^−1^ during the initial growth stage to a maximum of 5.81 mm day^−1^ during the mid-development stage of wheat. The average seasonal crop water requirement of wheat was determined as 427.28 mm. Thus, on average 4273 m^3^ of water is required per hectare of land to fully grow kingbird wheat variety in the study area. The relatively low wheat water requirement during the initial growth phase is due to the fact that little water is needed for seed germination and root system establishment. However, as the plant advances into the mid-season growth stage, marked by processes like stem elongation, budding, flowering, and grain filling, its water need increases significantly [28]. Furthermore, the fluctuation in crop water demand between the two growing seasons can be attributed to the variability in seasonal weather parameters. The elevated temperatures, increased wind speed, extended sunshine hours, and reduced humidity observed during the 2021/22 growing season contributed to an elevated water requirement compared to the 2022/23 growing season.

**Table 5:** Stage wise and seasonal water requirements of Kingbird wheat variety.

Growing season	Initial (mm day^−1^)	Development (mm day^−1^)	Mid-season (mm day^−1^)	Maturity (mm day^−1^)	Seasonal (mm)	Seasonal (m^3^ ha^−1^)
2021/22	2.06	4.31	6.37	5.40	469.11	4691.1
2022/23	1.74	3.93	5.26	3.29	385.46	3854.6
Average	1.90	4.12	5.82	4.35	427.29	4272.9

### 3.3 Reference evapotranspiration

The average daily ETo computed for the study period are shown in Fig. 6, Fig. 7. The ETo value varied between 2.47 and 6.94 mm day^−1^ with an average value of 5.49 mm day^−1^ during 2021/22 growing season, while it varied between 2.57 and 5.27 mm day^−1^ with an average value of 4.32 mm day^−1^ during 2022/23 growing season. The pooled ETo values observed at each growth stages were 4.71, 4.59, 5.01 and 5.29 mm day^−1^, respectively during the initial, development, mid-season and late season stages (Table 6). The seasonal ETo value during the whole growth season was 471.30 mm. The higher ETo occurred during 2021 growing season signifies more environmental demand as a result of warmer and drier weather condition (Table 4).

**Table 6:** ETo during each wheat growth stage and growth seasons.

Growing season	Initial (mm day^−1^)	Development (mm day^−1^)	Mid-season (mm day^−1^)	Maturity (mm day^−1^)<	Seasonal (mm)
2021/22	5.05	5.06	5.41	6.47	519.56
2022/23	4.37	4.11	4.61	4.11	423.04
Average	4.71	4.59	5.01	5.29	471.30

The ETo values consistently surpass the actual wheat ETc during the majority of growth stages, except the mid-growth stage. This phenomenon can be ascribed to several factors, including variations in crop type, growth stage, canopy cover, and soil conditions, leading to disparities with a well-irrigated, hypothetical reference crop usually grass under standardized meteorological conditions.

### 3.4 Crop coefficient of wheat

**Fig. 8:**
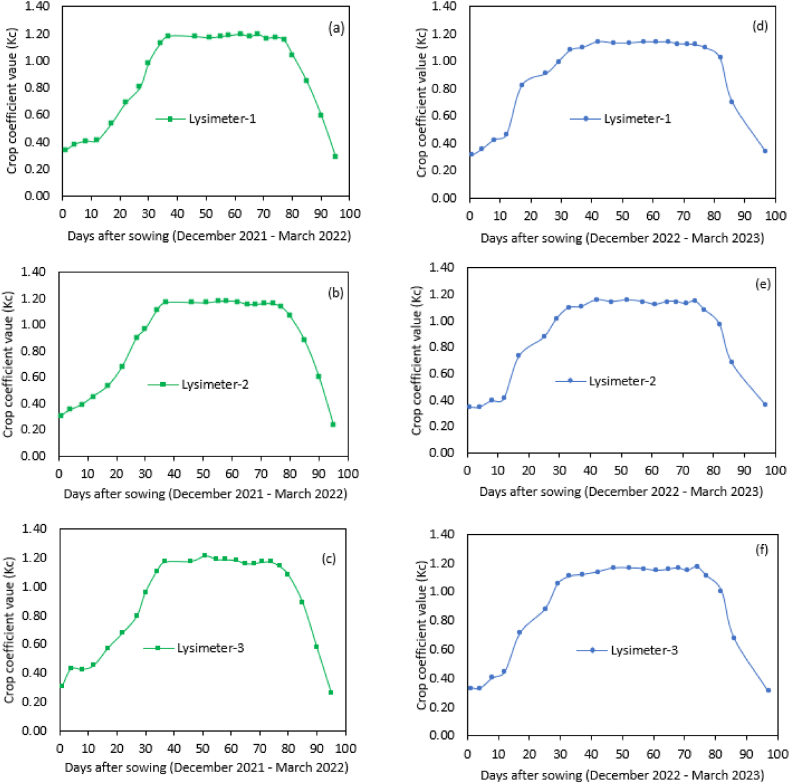
Observed crop coefficient values for wheat across each lysimeter: (a), (b) and (c) during 2021-22 growing season and (d), (e) and (f) during 2022-23 growing season.

The values of Kc for Kingbird wheat variety were computed and presented in Table 7 and Fig. 8. The average Kc values ranged from minimum of 0.43 in the initial stage to maximum of 1.15 at mid-season, and then decreased to 0.30 when the wheat ripened during the late-season growth stage. The increase in Kc value during the development and mid-season stages is due to the rapid crop development. As the crop develops and shades the ground to the effective full cover and reach full size with increasing plant height and root depth, the amount of water abstraction increased which in turn increased the ETc [4,26].

**Table 7:** Kingbird wheat variety Kc values at different stages of growing season.

Growing year		Growing stages		
	Initial	Development	Mid-season	End of season
2021/22	0.42	0.89	1.17	0.26
2022/23	0.43	0.98	1.13	0.34
Average	0.43	0.93	1.15	0.30

The wheat crop coefficient curves for each lysimeter are presented in Fig. 8. A consistent increase in Kc is noticeable from the initial stage to the mid-growth stage across all lysimeter plots, though with minor fluctuations. This upward trend in Kc indicates an elevated water demand during the active growth phases of the wheat crop. Notably, during the critical period spanning approximately from 35 to 80 days after sowing (DAS), the Kc values reached their maximum. This heightened Kc during this stage may signify a peak in the water requirement, possibly aligning with rapid vegetative growth and early reproductive development.

Subsequently, in the maturity stage, there was a gradual decline in Kc values for the wheat. This decline aligns with the typical developmental progression of the crop, where the demand for water diminishes as the crop approaches maturity. The decreasing Kc values during the maturity stage suggest a reduced reliance on water resources as the wheat crop undergoes its final growth phase, transitioning towards grain filling and maturation [4].

Similar experimental studies have been conducted in various regions in the world to determine Kc values for wheat. Based on these studies, the average Kc values in the initial, development, mid-season and end of season growth stages are 0.56, 0.92, 1.12 and 0.73 (Table 8), respectively. These values are close to the Kc value obtained in this study. The slightly variation at the initial and late growth stage in this study could be due to the crop variety, local physical and climatic conditions.

**Table 8:** The crop coefficient (Kc) of winter wheat under different irrigation methods in different regions from the initial growth period stage to the late growth stage.

Sources	Initial growth stage	Development stage	Mid-stage	End of growth stage
Feng et al. [29]	0.77	1.09	1.14	0.7
Amdneh and Ayana [14]	0.51	0.83	1.29	0.52
Yang et al. [30]	0.60	0.88	1.07	0.72
Li et al. [31]	0.66	0.89	1.01	0.88
Ketema et al. [13]	0.54	–	1.15	0.67
Kenjabaev et al. [32]	0.27		1.03	0.89
Mean	0.56	0.92	1.12	0.73
This study	0.43	0.93	1.15	0.30

**Fig. 9:**
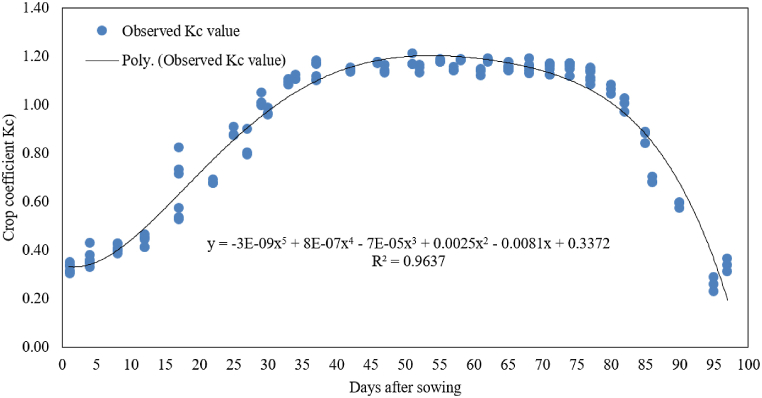
Relationship between crop coefficient (Kc) and days after sowing for wheat.

A fifth-order polynomial equation, developed using regression analysis, exhibited a high coefficient of determination (R^2^) and accurately predicted Kc values for Kingbird wheat variety as a function of growing days under semi-arid conditions in Melkassa (Fig. 9). A comparable fifth-order model was suggested for winter wheat in the semi-arid regions of China [30].

The actual Kc values obtained from the study were compared with those provided by Allen et al. [4], using their calibration methodology to adjust for local climatic conditions such as humidity and wind speed. The standard Kc values for wheat from Allen et al. [4] were 0.30 for the initial stage, 1.15 for the mid-stage, and 0.40 for the end of the season. The adjusted Kc values were 0.43, 1.15, and 0.46 for the initial, mid-stage, and end of the season, respectively.

For the initial growth stage, the calibrated Kc value (0.43) perfectly matched the actual field measurement (0.43), demonstrating the accuracy of the calibration methodology in capturing local climatic influences. During the mid-stage, both the standard and calibrated Kc values (1.15) matched the actual field measurement (1.15), indicating that the mid-stage Kc value is robust across different conditions. For the end of the season, the calibrated Kc value (0.46) was higher than the actual field measurement (0.30). This discrepancy suggests a divergence in water consumption patterns during the late growth stage, emphasizing the need for the development of local Kc values for local crop varieties and under actual field conditions.

## 4 Conclusion

The study offers useful observation into the dynamics of evapotranspiration, components of water balance, crop water requirements, and crop coefficient for Kingbird variety wheat in the semi-arid area of Melkassa. The daily average wheat evapotranspiration ranged from 1.90 mm day^−1^ during the initial stage to 5.81 mm day^−1^ at mid-season. The daily average wheat evapotranspiration ranged from 1.90 mm day to 1 during the initial stage to 5.81 mm day^−1^ at mid-season. The average seasonal crop water requirement was determined to be 427.28 mm, indicating that 4273 m^3^ of water are needed for the production of wheat per hectare. Crop coefficient values were 0.43, 1.15, 0.93, and 0.30 for the initial, development, mid-season, and late season stages. The study also presented a fifth-order polynomial equation that accurately predicts Kc values. While these values deviate slightly from the FAO-adjusted values for the study area, this highlights the need for localized calibration of crop coefficients, especially for specific cultivars like Kingbird. These findings are significant for Ethiopian agriculture, addressing gaps in wheat water requirements and crop coefficients, and can improve irrigation management practices in Ethiopia.

## Data availability statement

The data associated with our study has not been deposited into a public available repository. However, the data used in this study can be made available upon reasonable request to the corresponding author.

## CRediT authorship contribution statement

**Gobena Dirirsa Bayisa:** Writing – review & editing, Writing – original draft, Methodology, Formal analysis, Data curation, Conceptualization. **Mekonen Ayana:** Writing – review & editing, Validation, Supervision, Methodology, Investigation, Conceptualization. **Boja Mekonnen:** Writing – review & editing, Validation, Methodology. **Tilahun Hordofa:** Writing – review & editing, Validation, Supervision, Methodology, Data curation, Conceptualization. **Megersa Olumana Dinka:** Writing – review & editing, Conceptualization.

## Declaration of competing interest

The authors declare that they have no known competing financial interests or personal relationships that could have appeared to influence the work reported in this paper.
